# A sensitive mass spectrometry-based method to identify common respiratory pathogens in children

**DOI:** 10.1128/spectrum.01858-23

**Published:** 2023-09-27

**Authors:** Lixin Hu, Shenyan Zhang, Wenqi Song, Fang Dong, Zhengde Xie, Xiangpeng Chen, Meng Liu, Baoxue Cui, Yunheng Zhang, Rui Zhang, Qingtao Wang

**Affiliations:** 1 Capital Medical University, Beijing, China; 2 Department of Clinical Laboratory, Beijing Chao-Yang Hospita, Capital Medical University, Beijing, China; 3 Department of Clinical Laboratory, Beijing Children’s Hospital, Capital Medical University, National Center for Children’s Health, Beijing, China; 4 Beijing BGI-GBI Biotech Co., Ltd., Beijing, China; 5 BGI Genomics, Shenzhen, China; University of Maryland School of Medicine, Baltimore, Maryland, USA

**Keywords:** children common respiratory pathogen, matrix-assisted laser desorption/ionization time of flight mass spectrometry, pediatric respiratory tract infections, multiplex reverse transcription-PCR, multipathogen infections

## Abstract

Public health threats posed by emerging respiratory infections are a significant concern, particularly in children and infants. Traditional culture-based detection methods are time-consuming and typically require 1–3 days. Herein, we developed and evaluated a 23-plex common respiratory pathogen mass spectrometry assay that enables the simultaneous detection of 18 common respiratory pathogens in children. This assay combines matrix-assisted laser desorption/ionization time of flight mass spectrometry with multiplex reverse transcription-PCR and targets 11 bacterial and 7 viral pathogens (including 10 subtypes), and two internal controls. The detection limit of the common respiratory pathogen mass spectrometry assay was as low as 1 copy/µL, with no cross-reactivity with other organisms. We assessed the clinical performance of the common respiratory pathogen mass spectrometry assay using respiratory samples from 450 children. The total 450 clinical specimens underwent analysis via matrix-assisted laser desorption/ionization time of flight mass spectrometry, and the outcomes were juxtaposed with those derived from real-time reverse-transcriptase PCR conducted concurrently. The concordance between these methods was 96.0%, and the multiple infection identification rate was 7.1%. This innovative approach enables the simultaneous analysis of numerous outcomes from a solitary examination across 192 specimens within a timeframe of approximately 7 hours, with a dramatically reduced sample use and cost. In summary, the common respiratory pathogen mass spectrometry assay is a sensitive, accurate, and cost-effective method for detecting common respiratory pathogens in children and has the potential to revolutionize the diagnosis of respiratory tract infections.

**IMPORTANCE:**

This study aimed to present and evaluate a novel co-detection method that enables the simultaneous identification of 11 bacterial and 7 viral pathogens in about 7 hours using matrix-assisted laser desorption/ionization time of flight mass spectrometry. Our approach utilizes a combination of multiplex reverse transcription-PCR and matrix-assisted laser desorption/ionization time of flight mass spectrometry, which overcomes the limitations of conventional assays, which include a long assessment time, technical difficulty, and high costs. As a screening method for common respiratory pathogens in children, common respiratory pathogen mass spectrometry assay has the potential to revolutionize the diagnosis of respiratory tract infections by providing an accurate etiological diagnosis. The common respiratory pathogen mass spectrometry assay is expected to be a critical tool for the diagnosis of respiratory infections in children, offering a more efficient, cost-effective, and accurate approach for the detection of common respiratory pathogens.

## INTRODUCTION

Acute respiratory tract infections (ARTIs) are caused by various pathogens, including bacteria and viruses. ARTIs are responsible for millions of hospitalizations and deaths annually, making them a leading cause of morbidity and mortality in children under 5 years of age worldwide, resulting in 4 million deaths yearly (1 – 3). Currently, the most common methods for detecting and diagnosing ARTIs include culturing, serological testing (4), and techniques pertaining to molecular biology. Although standard pathogen culture protocols have been the primary tools for detecting bacteria and viruses in clinical laboratories, these methods are associated with long processing times, which usually range from 1 to 3 days, complex procedures, low sensitivity, and high costs (5). In contrast, molecular biology techniques, including PCR are rapid and sensitive for the diagnosis of pathogens. Furthermore, a multitude of nascent technologies, including multiplex real-time PCR and microarray methodologies, have become accessible for employment in clinical settings (6). However, the throughput of these reactions is insufficient. Despite advancements in methods such as GeneChip and high-throughput sequencing technology, the requirements for sequencing and cost limit their use in large-scale experiments (7, 8).

The clinical presentation of ARTIs caused by various pathogens can be similar, making it challenging to distinguish among them (9). Therefore, early definitive pathogen identification is crucial for clinical decision-making and poses significant diagnostic and therapeutic challenges. In recent decades, the detection of pathogens has been facilitated through the utilization of matrix-assisted laser desorption/ionization time of flight mass spectrometry (MALDI-TOF MS). This technique involves the use of multiplex PCR to amplify genes containing the target pathogens, followed by the utilization of single-base extension (SBE). Ultimately, MALDI-TOF MS is employed to identify the mass-to-charge ratio (*m*/*z*) of the SBE, so as to achieve the purpose of detecting pathogens. With massive technological advances in MALDI-TOF MS, precision medicine and public health can now be used (10).

Herein, we utilized a combination of MALDI-TOF MS (Beijing BGI-GBI Biotech Co., Ltd., Beijing, China) and multiplex reverse transcription-PCR (MRT-PCR) to detect and identify 18 common respiratory bacteria and viruses in children, including 11 bacterial pathogens and 7 viruses. Given the numerous subtypes of viruses, the 18 pathogens were divided into two wells: well 1, comprising 17 assays [*Haemophilus influenzae* (HIN), *Pseudomonas aeruginosa* (PAE), *Staphylococcus aureus* (SA), *Klebsiella pneumoniae* (KPN), *Escherichia coli* (ECO), *Acinetobacter baumannii* (ABA), *Moraxella catarrhalis* (MC), *Streptococcus pyogenes* (SPY), *Stenotrophomonas maltophilia* (SMA), *Respiratory syncytial virus type* A (RSVA), *Adenovirus type* C (ADVC), *Adenovirus type E* (ADVE), *Influenza A virus* (IFA), *Influenza B virus* (IFB), *Parainfluenza virus type 1* (PIV1), *Parainfluenza virus type 3* (PIV3), Glyceraldehyde-3-phosphate dehydrogenase 2 (GAPDH2)] and well 2, comprising six assays [*Streptococcus pneumoniae* (SPN), *Enterobacter cloacae* (ECL), *Respiratory syncytial virus type B* (RSVB), *Adenovirus type B* (ADVB), *Parainfluenza virus type 2* (PIV2), Glyceraldehyde-3-phosphate dehydrogenase 1 (GAPDH1)]. This approach can serve as a powerful complement to the diagnosis of respiratory tract infections.

## RESULTS

### Establishment and optimization of the common respiratory pathogen MS method

**Fig 1:**
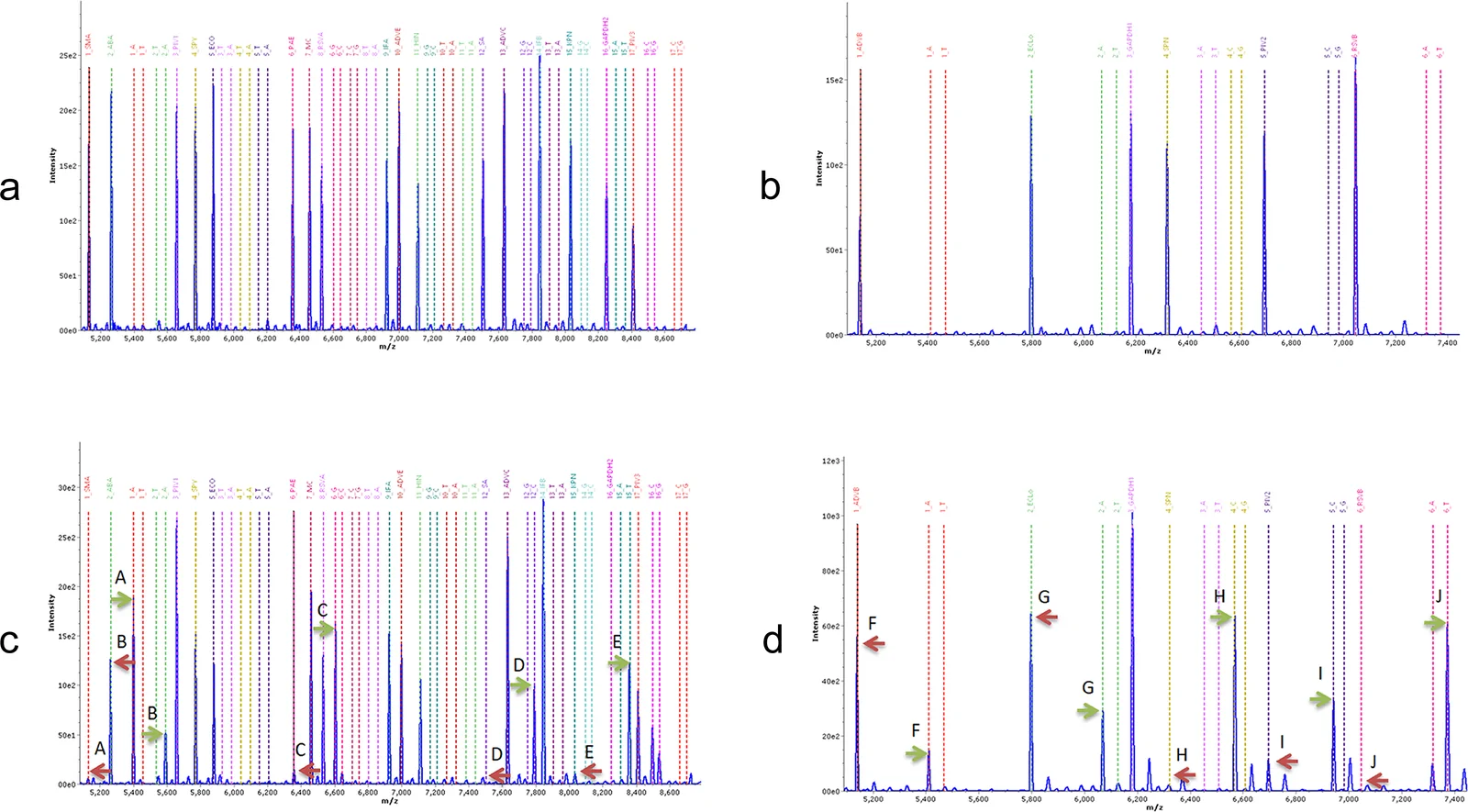
Common respiratory pathogen mass spectrometry (CCRP-MS) peak of the SBE primer. (a) CCRP-MS peaks of 17 SBE primers without extension in well 1; (b) CCRP-MS peaks of six SBE primers without extension in well 2; (c) target site peaks of the SBE primers extended to five pathogens in well 1 [containing (A) SMA, *Stenotrophomonas maltophilia*; (B) ABA, *Acinetobacter baumannii*; (C) PAE, *Pseudomonas aeruginosa*; (D) SA, *Staphylococcus aureus*; (E) KPN, *Klebsiella pneumoniae*]; and (d) target site peaks of the SBE primers extended to five pathogens in well 2 [containing (F) ADVB, *Adenovirus type B*; (G) ECL, *Enterobacter cloacae*; (H) SPN, *Streptococcus pneumoniae*; (I) PIV2, *Parainfluenza virus type 2*; (J) RSVB, *Respiratory syncytial virus type B*]. Red arrows indicate the SBE primer peaks disappearing or diminishing, whereas green arrows indicate the product peaks appearing.

In this study, the 18 target pathogens were amplified using 23 primers, and GAPDH1 and GAPDH2 were used as internal controls. All amplified products were verified by plasmid analysis. Positive results were indicated by the appearance of product peaks and disappearance or reduction of single-base extension primer peaks (Fig. 1).

### Evaluation of the sensitivity and specificity of the CCRP-MS method

**Fig 2:**
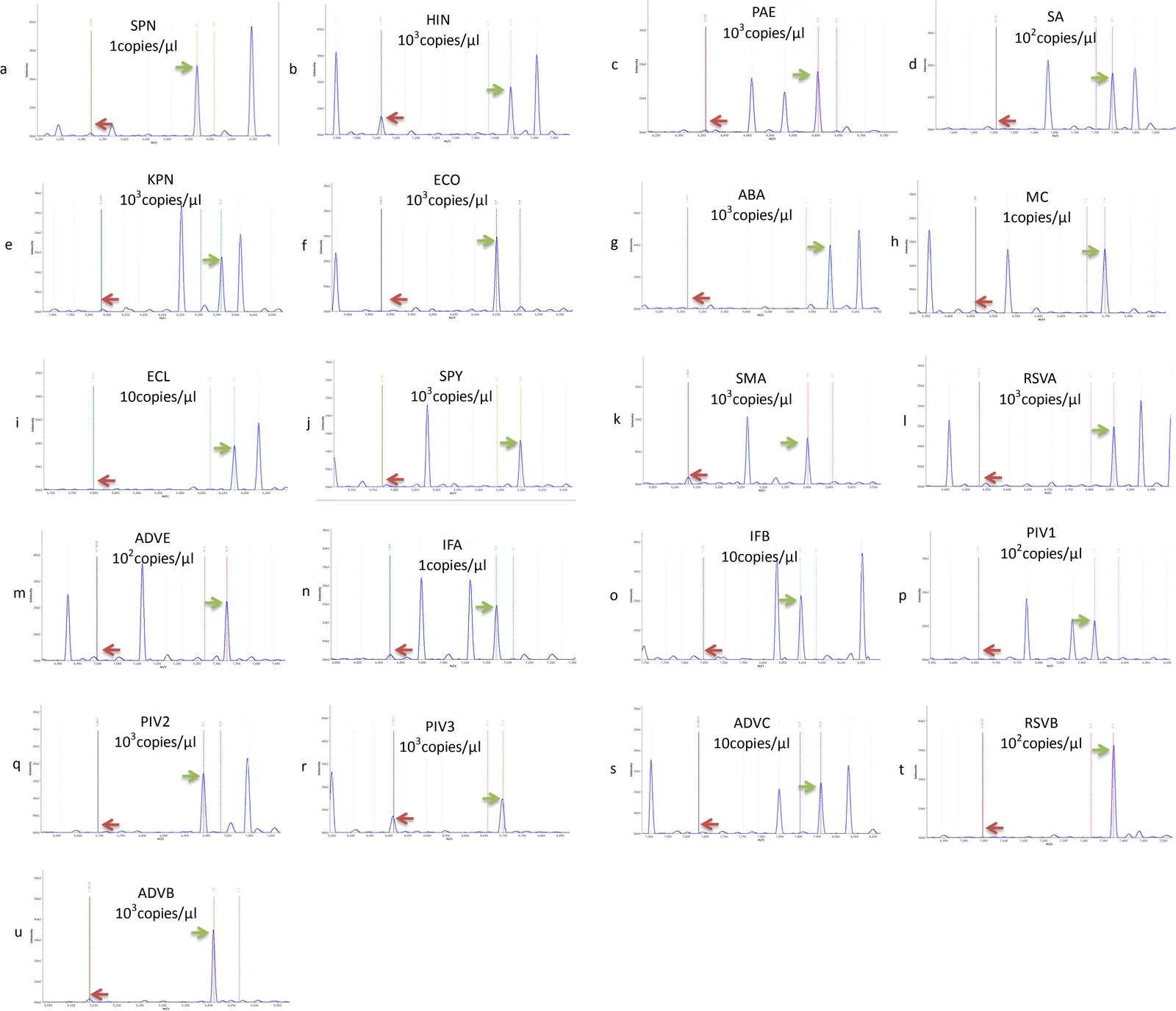
LOD of CCRP-MS: (a) SPN, *Streptococcus pneumoniae*; (b) HIN, *Haemophilus influenzae*; (c) PAE, *Pseudomonas aeruginosa*; (d) SA, *Staphylococcus aureus*; (e) KPN, *Klebsiella pneumoniae*; (f) ECO, *Escherichia coli*; (g) ABA, *Acinetobacter baumannii*; (h) MC, *Moraxella catarrhalis*; (i) ECL, *Enterobacter cloacae*; (j) SPY, *Streptococcus pyogenes*; (k) SMA, *Stenotrophomonas maltophilia*; (l) RSVA, *Respiratory syncytial virus type A*; (m) ADVE, *Adenovirus type E*; (n) IFA, *Influenza A virus*; (o) IFB, *Influenza B virus*; (p) PIV1, *Parainfluenza virus type 1*; (q) PIV2, *Parainfluenza virus type 2*; (r) PIV3, *Parainfluenza virus type 3*; (s) ADVC, *Adenovirus type C*; (t) RSVB, *Respiratory syncytial virus type B*; and (u) ADVB, *Adenovirus type B*. Red arrows indicate the single-base extension primer peaks disappearing or diminishing, whereas green arrows indicate the product peaks appearing.

In this study, the limit of detection (LOD) ranged from 1 to 10^3^ copies/μL. A comprehensive illustration of the LOD is shown in Fig. 2. To assess the specificity of the developed method, we conducted an experiment in which 10 plasmids or nucleic acids were mixed in wells 1 and 2 and subjected to the detection procedure. It is noteworthy that none of the mixed samples produced affirmative outcomes. The plasmids tested did not exhibit any cross-reactivity. A detailed analysis of the results is provided in Fig. S1.

### Clinical verification of the CCRP-MS method

In this study, 450 clinical samples were analyzed using both common respiratory pathogen mass spectrometry (CCRP-MS) and RT-PCR to detect 18 pathogens. The overall agreement rate between both methods was 96% (*n* = 432), whereas the discordance rate was 4% (*n* = 18). Discordant results were subjected to sequencing for further confirmation. The positive ratios for CCRP-MS and RT-PCR were 84.7% and 83.3%, respectively. The two testing methods had excellent agreement [kappa, 0.851; 95% confidence interval (CI), 0.784–0.918]. Table 1 presents a summary of the comparison of the clinical samples analyzed using both methods and Table 2 validates the discordance between the results of both methods.

**TABLE 1:** Comparison of the clinical samples analyzed using CCRP-MS and RT-PCR^*a*^

CCRP-MS	RT-PCR		Total
	Positive	Negative	
Positive	369	12	381
Negative	6	63	69
Total	375	75	450

^*a*^ Kappa, 0.851; 95% CI, 0.784–0.918; the two testing methods showed excellent agreement.

**TABLE 2:** Confirmation of the discordance between the results of CCRP-MS and RT-PCR

Samples	CCRP-MS result	RT-PCR result	Sequencing results
1	*Streptococcus pneumoniae*	–^*a*^	*Streptococcus pneumoniae*
2	*Enterobacter cloacae*	–	*Enterobacter cloacae*
3	*Streptococcus pneumoniae*	–	*Streptococcus pneumoniae*
4	*Streptococcus pneumoniae*	–	*Streptococcus pneumoniae*
5	*Streptococcus pneumoniae*	–	*Streptococcus pneumoniae*
6	*Streptococcus pneumoniae*	–	*Streptococcus pneumoniae*
7	*Enterobacter cloacae*	–	*Enterobacter cloacae*
8	*Streptococcus pneumoniae*	–	*Streptococcus pneumoniae*
9	–	*Haemophilus influenzae*	*Haemophilus influenzae*
10	–	*Influenza A virus*	*Influenza A virus*
11	–	*Influenza A virus*	*Influenza A virus*
12	–	*Parainfluenza virus type 1*	*Parainfluenza virus type 1*
13	*Parainfluenza virus type 1*	–	*Parainfluenza virus type 1*
14	*Parainfluenza virus type 3*	–	*Parainfluenza virus type 3*
15	–	*Parainfluenza virus type 3*	*Parainfluenza virus type 3*
16	–	*Adenovirus*	*Adenovirus*
17	*Respiratory syncytial virus*	–	*Respiratory syncytial virus*
18	*Respiratory syncytial virus*	–	*Respiratory syncytial virus*

^*a*^ –, negative result.

## DISCUSSION

**Fig 3:**
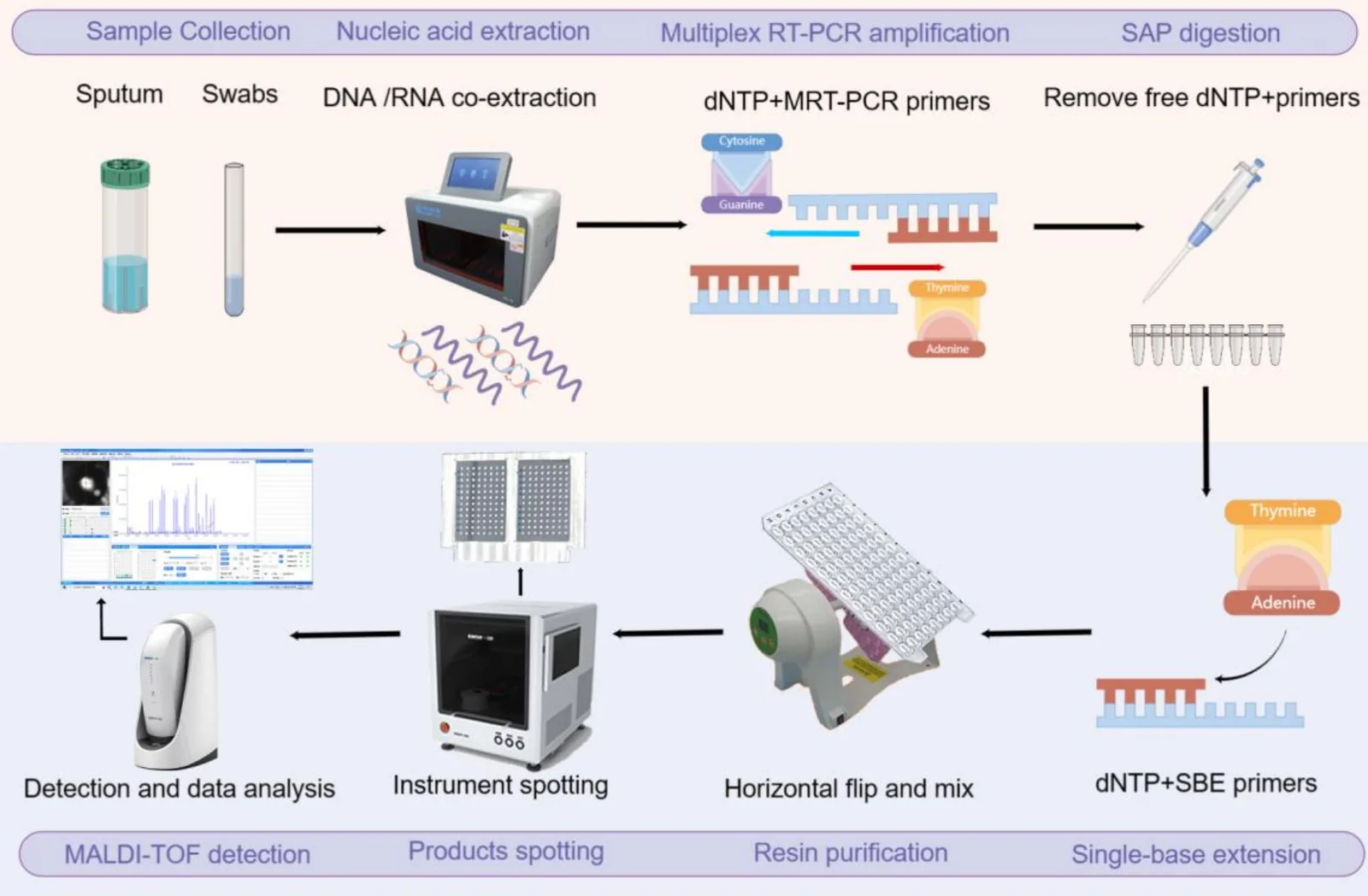
The flowcharts of CCRP-MS method for multiple pathogens detection in about 7 hours.

In the pediatric population worldwide, the incidences of hospitalization and fatalities due to severe acute lower respiratory infections (ALRI) remain to be elucidated (11). Given that ALRI is the leading cause of death among children aged 1–5 years worldwide, there is an urgent need to better understand the association between pathogens and ALRI (12). Infections that necessitate watchful waiting or acute respiratory tract infections are the most prevalent reasons for prescribing antibiotics to children under 14 years of age (13). Advancements in molecular biology and high-throughput technologies have enabled the rapid examination of multiple pathogens. Herein, we describe the development of a 23-plex approach for identifying common respiratory pathogens in children. Figure 3 demonstrates the flowchart pertaining to this method, which may aid clinicians in making etiological diagnoses. Additionally, this technique can be employed in parallel with other techniques for the multiplex detection of several pathogens.

Respiratory viruses and bacteria often cause lower airway infections, leading to increased airway inflammation (14). Compared to traditional identification methods, our approach offers significant advantages, including high sensitivity (ability to detect as low as 1 copy/μL) and accuracy. Compared to other molecular biology techniques, including multiplex PCR, gene chip, sequencing technology, our approach, which utilizes CCRP-MS, offers a notable benefit in terms of high throughput. This allows for the concurrent identification of 18 pathogens in a single experiment, at a low cost of 192 samples. In addition, our methodology is scalable and extensible, further enhancing its utility.

Notably, we identified 12 samples that tested positive using CCRP-MS but negative using RT-PCR and confirmed these results with Sanger sequencing, suggesting that CCRP-MS offers higher sensitivity and specificity than RT-PCR. However, we also observed six samples that tested positive using RT-PCR and Sanger sequencing but were negative using our method, which may be attributed to various factors, including the different experimental designs of CCRP-MS and RT-PCR; the high throughput of the test in well 1 may highly increase the possibility of mutual interference between the use of detection reagents, which will reduce the efficiency of the reaction or suboptimal reaction conditions. Additionally, residual salt and organic solvents from nucleic acid extraction may inhibit multiple amplification efficiencies, and repeated freeze-thaw cycles or enzyme inactivation may lead to false-negative results.

This technology is capable of detecting not only single viruses and bacteria but also multiplex respiratory pathogens. In our study, among the 450 clinical samples, the rate of multiple infections detected using CCRP-MS was 7.1% (Table S4), which is consistent with the results of the study of Jain et al. (15). The increasing use of panel-based multiplex pathogen testing for diagnosing respiratory has resulted in a rapid turnaround (16, 17). While some researchers have used the PCR-mass assay to address the inherent limitations of simultaneously detecting various respiratory viruses using MALDI-TOF MS (18 – 21), the novelty of our method lies in two aspects: (i) the co-detection of common bacterial and viral agents, and (ii) the combination of MRT-PCR and MALDI-TOF MS technology to detect common pathogens in children’s respiratory tract samples.

However, it is important to note that CCRP-MS is only capable of the qualitative identification of pathogens but not of quantitative detection. Clinicians must make judgments based on all available information. Although we analyzed 450 clinical samples, the sample size remained limited, and further studies are required to validate the clinical utility of CCRP-MS. CCRP-MS, a 23-plex assay combining MALDI-TOF MS with MRT-PCR, can be used to directly detect common respiratory pathogens in children. We believe that this method can complement the existing methods and contribute to the clinical laboratory diagnosis of respiratory tract infections in children.

## MATERIALS AND METHODS

### Extraction of clinical samples and nucleic acid

A total of 450 samples were collected from children with respiratory symptoms at the time of clinical examination at the Beijing Children’s Hospital, Capital Medical University. The sample types included oropharyngeal and nasopharyngeal swabs, tracheal secretions, bronchial secretions, and sputum. Some samples, including sputum, tracheal, and bronchial secretions, were viscous. A liquefying agent (BASO BC1997) was used to obtain a homogeneous solution. DNA and RNA were directly extracted from 200 µL of each sample using the EX-48 Automated Nucleic Acid Extraction System (Beijing BGI-GBI Biotech Co., Ltd., Beijing, China). The ethical review board of the Beijing Children’s Hospital Capital Medical University deemed that this study did not require the provision of informed consent.

### CCRP-MS study design

Target gene sequences of the pathogens were downloaded from the National Center for Biotechnology Information database (https://www.ncbi.nlm.nih.gov/), and at least 300 sequences were obtained for each pathogen to ensure that they were representative. MAFFT was used to align multiple sequences and select highly homologous sequences as candidates for primer design (https://www.ebi.ac.uk/Tools/msa/mafft/). Primers and single-base extension primers for multiplexed assays were designed using the MassARRAY Assay Design 3.1 software (Agena Bioscience, Inc., San Diego, CA, USA) (Table 3). GAPDH was used as an internal control, and the target specificity of the primers and probes was checked using the Basic Local Alignment Search Tool (BLAST) (https://blast.ncbi.nlm.nih.gov/Blast.cgi). All primers were synthesized by BGI Tech Solutions (Beijing Liuhe Greatness Technology Co., Ltd., Beijing, China).

**TABLE 3:** Primer sequences in this study

Pathogen^*a*^	Target gene	Forward primer	Reverse primer	Single-base extension primers
SPN	Lyta	ACGTTGGATGTCGACAACTCAGGCGAAATG	ACGTTGGATGTCATGGCACCTTCTTCGTTG	CACTTATCAGCGATTTTCTTC
HIN	Fuck	ACGTTGGATGTTCTCAAGGCTTAACCACTG	ACGTTGGATGATTCTATGACGCCAGAACCC	GCCGCTGGATTAAAGCAATTGGA
PAE	Gyrb	ACGTTGGATGTACGTGCAGAAGGGTGAGC	ACGTTGGATGTTGGTACCTTCGGCCATCAG	CCCCTCCTTGAAGGCGCTGAT
SA	Nuc	ACGTTGGATGTTAGCCAAGCCTTGACGAAC	ACGTTGGATGGAAGTCGAGTTTGACAAAGG	GGGGAACTGATAAATATGGACGTG
KPN	Glta	ACGTTGGATGGGCGTATTTACCTTTGACCC	ACGTTGGATGCCTCGTCACCATCGATAAAC	GGGATTTTAGATTCACAAGAAGCCGT
ECO ABA MC ECL	UidA OXA Copb Ompx	ACGTTGGATGTCGTTAAAACTGCCTGGCAC ACGTTGGATGAAGTTAAGGGAGAACGCTAC ACGTTGGATGACCATTACCACCGCCAAAAC ACGTTGGATGTGGACTTCTCCTATGAGCAG	ACGTTGGATGGTGGAATTGATCAGCGTTGG ACGTTGGATGGATGTAGACCCACAAGTAGG ACGTTGGATGAAAGACGAAAGCACGGCTAC ACGTTGGATGTAGAAGCGGTAACCTACGCC	GAAAAGCGCGTTACAAGAA TAGGCTGGTTAACTGGA GGTTTGTTACAAGATGAACCT CGCGATCCAGGTGCCAACG
SPY SMA RSVA ADVE IFA IFB PIV1 PIV2 PIV3 ADVC	Mf Smet MP Hexon MP NS1 HN HN HN Hexon	ACGTTGGATGAGATGAGTTAGGAAGGACGC ACGTTGGATGCGATCATCTCCAGCGTGGTA ACGTTGGATGAGTAGATCTTGGAGCTTACC ACGTTGGATGTGTTGCTAACTACGATCCAG ACGTTGGATGTGAAAAGAGGGCCTTCTACG ACGTTGGATGCCCAATTTGGTCAAGAGCAC ACGTTGGATGGCGTATTCATCAAACTTAATC ACGTTGGATGAGACCAGAGGAAGCATCAAG ACGTTGGATGTTGTAACTTGCTGTGCCAAC ACGTTGGATGTCTATTGGCGATAGAACCAG	ACGTTGGATGTGTCTAACACCGTAGCTACC ACGTTGGATGAAAGAGGACACCCAGGCAAC ACGTTGGATGTCTTCCATGGGTTTGATTGC ACGTTGGATGGAGTATCTGGAGTCTGCAAG ACGTTGGATGATCGTCAACATCCACAGCAC ACGTTGGATGGATAAAGTTCTTCCGTGACC ACGTTGGATGCCGGGTTTAAATCAGGATAC ACGTTGGATGCCGAACTGCCACAATTCTTG ACGTTGGATGGGGTCAGAAGGAAGATTAC ACGTTGGATGTCTGACATCTGGATCATAGC	CCTTCAACATTGGCATAAG AGCCTGCTTCCATGAAC GGGGTTTGATTGCAAATCGTG ACCGCAAGTCAACATTTTCTGTG TTCCGGTACTCTTCCCTCATAGA CTCTCCCGTGACCAGTCTAATTGTCT CACTTCCCTATATCTGCAC TGTTCTATGTTCAAGTATTCTT GGGAGGAAGGAAGATTACTTCTACTAG GGCTCATAGCTGTCTACAGCCTGAT
RSVB	MP	ACGTTGGATGTTTATGAGCAAGTCTGCTGG	ACGTTGGATGGCATCACTAACAATATGGG	CACTAACAATATGGGTGCCTATG
ADVB	Hexon	ACGTTGGATGAAGTAGGTGTCTGTTGCACG	ACGTTGGATGATGGGCATACATGCACATCG	CATGCACATCGCCGGAC
GAPDH1	/^*b*^	ACGTTGGATGAGGTTTTTCTAGACGGCAGG	ACGTTGGATGAGGTCATCCCTGAGCTGAAC	GAATGCCAACGTGTCAGTGG
GAPDH2	/	ACGTTGGATGGCTTCACCACCTTCTTGATG	ACGTTGGATGACTGCCAACGTGTCAGTGGT	TCCTACCAACGTGTCAGTGGTGGACCT

^*a*^ SPN, *Streptococcus pneumoniae*; HIN, *Haemophilus influenzae*; PAE, *Pseudomonas aeruginosa*; SA, *Staphylococcus aureus*; KPN, *Klebsiella pneumoniae*; ECO, *Escherichia coli*; ABA, *Acinetobacter baumannii*; MC, *Moraxella catarrhalis*; ECL, *Enterobacter cloacae*; SPY, *Streptococcus pyogenes*; SMA, *Stenotrophomonas maltophilia*; RSVA, *Respiratory syncytial virus type A*; RSVB, *Respiratory syncytial virus type B*; ADVB, *Adenovirus type B*; ADVC, *Adenovirus type C*; ADVE, *Adenovirus type E*; IFA, *Influenza A viru*s; IFB, *Influenza B virus*; PIV1, *Parainfluenza virus type 1*; PIV2, *Parainfluenza virus type 2*; PIV3, *Parainfluenza virus type 3*; GAPDH1, Glyceraldehyde-3-phosphate dehydrogenase 1; GAPDH2, Glyceraldehyde-3-phosphate dehydrogenase 2.

^*b*^ "/" indicates that there are no target genes in GAPDH1 and GAPDH2.

### CCRP-MS method establishment

The synthetic and diluted plasmids were used to establish the CCRP-MS method. Nuclease-free water was used as a negative control. The procedure comprised four steps. (i) For each MRT-PCR, the target gene was amplified using a MALDI-TOF MS universal nucleic acid detection kit for RNA. (ii) The product was subsequently subjected to shrimp alkaline phosphatase digestion to remove unincorporated primers and deoxy-ribonucleoside triphosphate. (iii) SBE reaction, which can detect the presence of a pathogen, was applied after shrimp alkaline phosphatase treatment. (iv) The product was purified using resin.

### MALDI-TOF MS analysis

A fully automated sample handling system spotted the purified products onto the chips, and detection was performed with MALDI-TOF MS. The Nutyper program (Beijing BGI-GBI Biotech Co., Ltd., Beijing, China) was used for data analysis. All MALDI-TOF MS instruments, software, and reagents were purchased from Beijing BGI-GBI Biotech Co., Ltd., Beijing, China.

### Sensitivity and specificity of CCRP-MS

A 10-fold gradient dilution was used to dilute the plasmids from 10^4^ copies/μL to 1 copy/μL and determine the sensitivity of CCRP-MS. We quantified the original concentrations using a Qubit version 2.0 fluorometer (Thermo Fisher Scientific Inc.). Each constituent of the dilution series underwent triplicate analysis, and the minimum detectable concentration for that assay was determined as the highest dilution at which all three replicates tested positive. To gauge system specificity, we mixed 10 plasmids or nucleic acids with equal volume spotting and detecting in wells 1 and 2.

### Clinical samples validation

We tested 450 clinical samples using both the CCRP-MS and RT-PCR methods. The RT-PCR assay was custom-designed and executed using the HiScript II One Step RT-PCR Kit (Vazyme Biotech Co., Ltd.) in accordance with established academic procedures. Results that were not in agreement were sent for gene sequencing for further confirmation.

## SUPPLEMENTAL MATERIAL

Fig. S1, Tables S1 to S4 spectrum.01858-23-s0001.docx A comprehensive illustration of the article.

ASM does not own the copyrights to Supplemental Material that may be linked to, or accessed through, an article. The authors have granted ASM a non-exclusive, world-wide license to publish the Supplemental Material files. Please contact the corresponding author directly for reuse.
