# Supplementary material for: A sensitive mass spectrometry-based method to identify common respiratory pathogens in children
Source: Microbiol Spectr. 2023 Sep 27;11(5):e01858-23. doi: 10.1128/spectrum.01858-23 (PMC10580997; doi:10.1128/spectrum.01858-23)
Supplement: Fig. S1, Tables S1 to S4 — A comprehensive illustration of the article. [file spectrum.01858-23-s0001.docx]

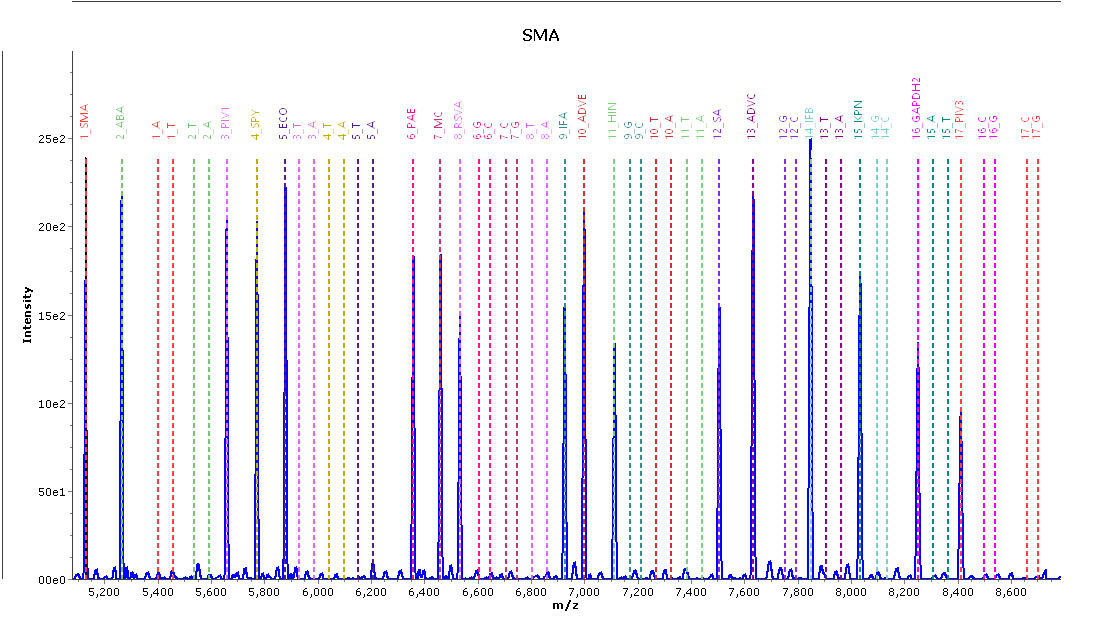


A


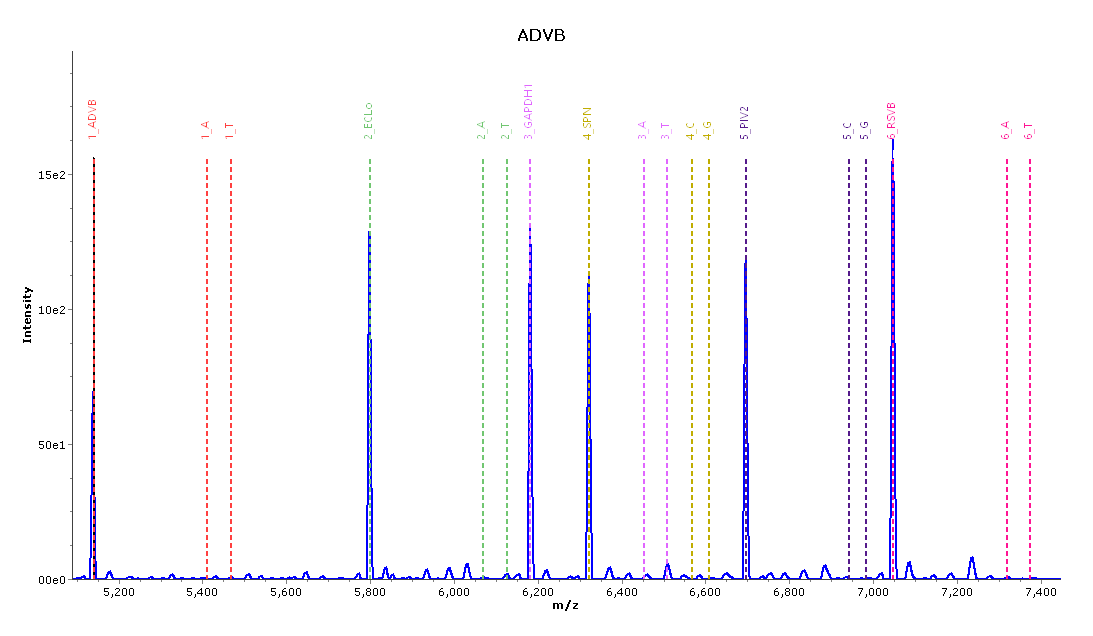


B

**Fig. S1** Specifically, we conducted a CCRP-MS analysis by combining 10 plasmids or nucleic acids and spotting them in equal volumes in well1 and well2, respectively. The samples contained *Hepatitis B virus, Coxsackie virus, Dengue Virus, Hantaan virus, Mycobacterium tuberculosis, Human papillomavirus, Neisseria gonorrhoeae, Staphylococcus epidermidis, Enterococcus faecium,* and *Candida albicans*. Well1 is indicated by A, while well2 is indicated by B. The results consistently demonstrated negativity.

**Table S1** Clinical samples in this study

| Pathogen^a^ | Number |
| --- | --- |
| SPN | 40 |
| HIN | 28 |
| PAE | 61 |
| SA | 56 |
| KPN | 15 |
| ECO  ABA  MC  ECL | 21  24  6  8 |
| SPY  SMA  RSV  ADV  IFA  IFB  PIV1  PIV2  PIV3  Total | 1  8  42  25  28  5  10  4  37  450 |

^a^SPN, *Streptococcus pneumoniae*; HIN, *Haemophilus influenzae*; PAE, *Pseudomonas aeruginosa*; SA, *Staphylococcus aureus*; KPN, *Klebsiella pneumoniae*; ECO, *Escherichia coli*; ABA, *Acinetobacter baumannii*; MC, *Moraxella catarrhalis*; ECL, *Enterobacter cloacae*; SPY, *Streptococcus pyogenes*; SMA, *Stenotrophomonas maltophilia*; RSV, *Respiratory syncytial virus*; ADV, *Adenovirus*; IFA, *Influenza A virus*; IFB, *Influenza B virus*; PIV1, *Parainfluenza virus type 1*; PIV2, *Parainfluenza virus type 2*; PIV3, *Parainfluenza virus type 3*.

**Table S2** Lower limit of detection (LOD) of CCRP-MS

| Pathogen^a^ | LOD(copies/μl) | Pathogen^a^ | LOD(copies/μl) |
| --- | --- | --- | --- |
| SPN  HIN  PAE  SA  KPN  ECO  ABA  MC  ECL  SPY  SMA | 1  10^3^  10^3^  10^2^  10^3^  10^3^  10^3^  1  10  10^3^  10^3^ | RSVA  ADVE  IFA  IFB  PIV1  PIV2  PIV3  ADVC  RSVB  ADVB | 10^3^  10^2^  1  10  10^2^  10^3^  10^3^  10  10^2^  10^3^ |

^a^SPN, *Streptococcus pneumoniae*; HIN, *Haemophilus influenzae*; PAE, *Pseudomonas aeruginosa*; SA, *Staphylococcus aureus*; KPN, *Klebsiella pneumoniae*; ECO, *Escherichia coli*; ABA, *Acinetobacter baumannii*; MC, *Moraxella catarrhalis*; ECL, *Enterobacter cloacae*; SPY, *Streptococcus pyogenes*; SMA, *Stenotrophomonas maltophilia*; RSVA, *Respiratory syncytial virus type A*; ADVE, *Adenovirus type E*; IFA, *Influenza A virus*; IFB, *Influenza B virus*; PIV1, *Parainfluenza virus type 1*; PIV2, *Parainfluenza virus type 2*; PIV3, *Parainfluenza virus type 3*; ADVC, *Adenovirus type C*; RSVB, *Respiratory syncytial virus type B*; ADVB, *Adenovirus type* B.

**Table S3** Comparison the clinical samples result of using CCRP-MS with RT-PCR

| Pathogens | Positive by CCRP-MS No. (%) | False negative by CCRP-MS No. (%) | False negative by RT-PCR  No. (%) | Positive by RT-PCR No. (%) | Contradictory by sequencing No. (%) |
| --- | --- | --- | --- | --- | --- |
| *Streptococcus pneumoniae* | 35（7.8） | 0 | 6（1.3） | 29（6.4） | 6（1.3） |
| *Haemophilus influenzae* | 20（4.4） | 1（0.2） | 0 | 21（4.7） | 1（0.2） |
| *Pseudomonas aeruginosa* | 62（13.8） | 0 | 0 | 62（13.8） | 0 |
| *Staphylococcus aureus* | 48（10.7） | 0 | 0 | 48（10.7） | 0 |
| *Klebsiella pneumoniae* | 13（2.9） | 0 | 0 | 13（2.9） | 0 |
| *Escherichia coli* | 20（4.4） | 0 | 0 | 20（4.4） | 0 |
| *Acinetobacter baumannii* | 25（5.6） | 0 | 0 | 25（5.6） | 0 |
| *Moraxella catarrhalis* | 7（1.6） | 0 | 0 | 7（1.6） | 0 |
| *Enterobacter cloacae* | 9（2.0） | 0 | 2（0.4） | 7（1.6） | 2（0.4） |
| *Streptococcus pyogenes* | 2（0.4） | 0 | 0 | 2（0.4） | 0 |
| *Stenotrophomonas maltophilia* | 8（1.8） | 0 | 0 | 8（1.8） | 0 |
| *Respiratory syncytial virus* | 42（9.3） | 0 | 2（0.4） | 40（8.9） | 2（0.4） |
| *Adenovirus* | 23（5.1） | 1（0.2） | 0 | 24（5.3） | 1（0.2） |
| *Influenza A virus* | 21（4.7） | 2（0.4） | 0 | 23（5.1） | 2（0.4） |
| *Influenza B virus* | 5（1.1） | 0 | 0 | 5（1.1） | 0 |
| *Parainfluenza virus type 1* | 8（1.8） | 1（0.2） | 1（0.2） | 8（1.8） | 2（0.4） |
| *Parainfluenza virus type 2* | 3（0.7） | 0 | 0 | 3（0.7） | 0 |
| *Parainfluenza virus type 3* | 30（6.7） | 1（0.2） | 1（0.2） | 30（6.7） | 2（0.4） |
| Total | 381（84.7） | 6（1.3） | 12（2.7） | 375（83.3） | 18（4.0） |

**Table S4** Multiple infections detected in both CCRP-MS and RT-PCR^a^

| Pathogens^b^ | No. |
| --- | --- |
| SA/PAE | 8 |
| SA/ECO | 4 |
| SA/MC | 2 |
| SA/PAE/KPN | 1 |
| SA/ECL | 1 |
| KPN/ECO | 1 |
| KPN/SMA | 1 |
| KPN/PAE | 1 |
| SMA/ECO | 1 |
| KPN/ECL | 1 |
| PAE/SPN | 1 |
| HIN/MC | 1 |
| PAE/ABA | 3 |
| HIN/ABA | 1 |
| SPN/MC | 1 |
| PAE/ ECO | 1 |
| ABA/ ECL | 1 |
| HIN/SPN | 1 |
| RSV/IFA | 1 |

^a^ The total number was 32.

**^b^** SA, *Staphylococcus aureus*; PAE, *Pseudomonas aeruginosa*; ECO, *Escherichia coli*; MC, *Moraxella catarrhalis*; KPN, *Klebsiella pneumoniae*; ECL, *Enterobacter cloacae*; SMA, *Stenotrophomonas maltophilia*; ABA, *Acinetobacter baumannii;* SPN, *Streptococcus pneumoniae*; HIN, *Haemophilus influenzae*; RSV, *Respiratory syncytial virus*; IFA, *Influenza A virus*.
